# Screening of Porcine Innate Immune Adaptor Signaling Revealed Several Anti-PRRSV Signaling Pathways

**DOI:** 10.3390/vaccines9101176

**Published:** 2021-10-14

**Authors:** Yulin Xu, Mengxue Ye, Youwen Zhang, Shaohua Sun, Jia Luo, Sen Jiang, Jiajia Zhang, Xueliang Liu, Qi Shao, Qi Cao, Wanglong Zheng, François Meurens, Nanhua Chen, Jianzhong Zhu

**Affiliations:** 1 College of Veterinary Medicine, Yangzhou University, Yangzhou 225009, China; ylxu15650096726@163.com (Y.X.); mengxueyzu@163.com (M.Y.); zyw18252734747@163.com (Y.Z.); shaohuas90@sina.com (S.S.); luojiajia20210518@163.com (J.L.); jiangsen8888888@163.com (S.J.); awaw0601@163.com (J.Z.); xueliangaaa@foxmail.com (X.L.); sq970123@163.com (Q.S.); qc_2021@163.com (Q.C.); 007297@yzu.edu.cn (W.Z.); 2 Joint International Research Laboratory of Agriculture and Agri-Product Safety, Yangzhou 225009, China; 3 Institute of Comparative Medicine Research Institute, Yangzhou University, Yangzhou 225009, China; 4 Jiangsu Co-Innovation Center for Prevention and Control of Important Animal Infectious Diseases and Zoonoses, Yangzhou 225009, China; 5 BIOEPAR, INRAE, Oniris, 44307 Nantes, France; francois.meurens@inra.fr; 6 Department of Veterinary Microbiology and Immunology, Western College of Veterinary Medicine, University of Saskatchewan, Saskatoon, SK S7N 5E2, Canada

**Keywords:** PRRSV, innate immunity, pattern recognition receptor (PRR), signaling adaptors, ectopic expression, agonist, knockdown

## Abstract

Porcine reproductive and respiratory syndrome virus (PRRSV) causes PRRS and is known to effectively suppress host innate immunity. The current strategies for controlling PRRSV are limited and complete understanding of anti-PRRSV innate immunity is needed. Here, we utilized nine porcine innate immune signaling adaptors which represent all currently known innate immune receptor signaling pathways for screening of anti-PRRSV activity. The analysis of PRRSV N gene transcription and protein expression both suggested that the multiple ectopic adaptors exhibited varying degrees of anti-PRRSV activities, with TRIF and MAVS most effective. To better quantify the PRRSV replication, the GFP signal of PRRSV from reverse genetics were measured by flow cytometry and similarly varying anti-PRRSV activities by different signaling adaptors were observed. Based on the screening data, and considering the importance of viral nucleic acid in innate immune response, endogenous TRIF, MAVS and STING were selected for further examination of anti-PRRSV activity. Agonist stimulation assay showed that MAVS and STING signaling possessed significant anti-PRRSV activities, whereas siRNA knockdown assay showed that TRIF, MAVS and STING are all involved in anti-PRRSV activity, with TLR3-TRIF displaying discrepancy in anti-PRRSV infection. Nevertheless, our work suggests that multiple pattern recognition receptor (PRR) signaling pathways are involved in anti-PRRSV innate immunity, which may have implications for the development of future antiviral strategies.

## 1. Introduction

Porcine reproductive and respiratory syndrome (PRRS) is one of the most widespread swine diseases in the world [1], characterized by reproductive failures in pregnant sows and respiratory diseases in piglets [2]. The pathogenic agent of PRRS, porcine reproductive and respiratory syndrome virus (PRRSV), belongs to the family *Arteriviridae* with a genome of positive-sense single-stranded RNA. PRRSV has a genome approximately 15.4 kb in length and is divided into two species (PRRSV1 and PRRSV2) based on the genomic sequence identity. The genome contains at least 11 open reading frames (ORFs), encoding 16 nonstructural proteins (nsp1-12) and 8 structural proteins [3]. The ORF1 including slightly overlapped ORF1a and ORF1b at the 5′ end of the viral genome accounts for about three-quarters of the entire genome and encodes 16 nonstructural proteins (nsp1α, nsp1β, nsp2, nsp2TF, nsp2N, nsp3-6, nsp7α, nsp7β, and nsp8-12). The 3′ end of the viral genome contains 8 ORFs (ORF2-ORF7) for viral structural proteins and the neighboring genes overlap with each other. The structural genes encode 4 membrane-associated glycoproteins (GP2a/ORF2a, GP3/ORF3, GP4/ORF4 and GP5/ORF5), 3 non-glycosylated membrane proteins (E/ORF2b, ORF5a and M/ORF6) and a nucleocapsid protein (N/ORF7) [2].

The innate immune system is the first line of defense against infections. Pathogen-associated molecular patterns (PAMPs) are recognized by host germline-encoded pattern recognition receptors, including Toll-like receptors (TLRs), RIG-I-like receptors (RLRs), NOD-like receptors (NLRs), C-type lectin receptors (CLRs), and cytosolic DNA receptors (CDRs) [4,5,6]. TLR signaling pathway is essentially divided into MyD88 and TRIF-dependent pathways based on the specific adaptor recruitment. The cell endosomal TLR3, TLR7, TLR8 and TLR9 appear to be sensors of foreign nucleic acids and trigger anti-viral innate immune responses by producing type I interferons (IFNs) and inflammatory cytokines [7]. RLRs RIG-I and MDA5 mainly recognize cytosolic viral RNA, and activate signaling adaptor MAVS, inducing type I IFNs and other antiviral molecules [8]. NLRs NOD1 and NOD2 utilize the adaptor RIPK2 to activate NF-κB signaling and induce proinflammatory mediators, whereas other NLR members activate adaptor ASC to induce inflammasome and mature IL-1 and IL-18 [9]. CLRs utilize the adaptor complex of CARD9-BCL10-MALT1 (CBM) to activate NF-κB signaling [10]. The recently identified CDR cGAS recognizes cytosolic DNA and activates adaptor STING, inducing type I IFNs and proinflammatory cytokines [11].

Type I IFNs (IFNα/β) are the most potent component of innate immunity fighting against invading viruses, including PRRSV [12]. However, PRRSV has evolved a number of mechanisms to evade type I IFN induction and signaling, suppressing type I IFN responses in both virus-infected cells and pigs [13]. Six PRRSV proteins; nsp1α, nsp1β, nsp2, nsp4, nsp11, and N, have been identified so far as IFN antagonists [14,15,16]. Specifically, nsp1α is able to degrade CREB-binding protein (CBP) in the nucleus and block the recruitment of co-factor CBP for IFN promoter activation and gene expression. Nsp1β inhibits the phosphorylation and nuclear translocation of transcription factor IRF3 and also block the nuclear translocation of ISGF3, thus suppressing not only IFN induction but also ISG induction. Nsp2 inhibits IFNβ production by suppressing the activation of the IRF-3 and NF-κB signaling via its DUB/OTU protease activity. Nsp4 appears to suppress IFNβ transcription by blocking NF-κB activation via its serine protease (SP) activity. Nsp11 inhibits the IRF3 and NF-κB signaling via its endoribonuclease (NendoU) activity, and it also impairs the formation of ISGF3 independent of its enzyme activity. The protein N inhibits IRF3 phosphorylation and nuclear translocation, and thus suppresses IFNβ induction.

Regardless of the IFN antagonism by PRRSV, some host factors have been identified as having antiviral functions against PRRSV, including interferon-induced protein with tetratricopeptide repeats 3 (IFIT3) [17], guanylate-binding protein 1 (GBP1) [18], myxovirus resistance protein 1 (Mx1) [19], zinc finger antiviral protein (ZAP) [20], NLRX1 [21], viperin [22], interferon gamma-inducible protein 16 (IFI16) [23] and cholesterol 25-hydroxylase (CH25H) [24]. Notably, all the host restriction factors are closely related with innate immunity, and all except NLRX1 are IFN-stimulated genes (ISGs). Therefore, there exists a delicate and intricate interplay between PRRSV and the innate immune system of it’s host. As an RNA virus, PRRSV has been assumed to be recognized by TLR3/7/8/9 and RLRs which trigger innate immune signaling [25], however, the global picture of interactions between PRRSV and innate immunity and the antiviral activities of different innate immune pathways have not been investigated before. Here we utilized nine porcine signaling adaptors reflecting all currently known innate immune signaling pathways to assess their anti-PPRSV activities.

## 2. Materials and Methods

### 2.1. Cells, Virus and Reagents

PRRSV-permissive Marc-145 and HEK293T cells were cultured in Dulbecco’s Modified Eagle’s Medium (DMEM; Life Technologies Corp, Grand Island, NE, USA) supplemented with 10% fetal bovine serum (FBS) and 100 U/mL of penicillin plus 100 μg/mL streptomycin. Porcine alveolar macrophages (3D4/21, ATCC CRL-2843) were cultured in RPMI (Hyclone Laboratories, Omaha, NE, USA) containing 10% FBS with penicillin/streptomycin. All cells were maintained at 37 °C with 5% CO_2_ in a humidified incubator. The PRRSV strain used in this study were highly pathogenic PRRSV XJ17-5 (PRRSV-2, GenBank ID: MK759853.1), which we isolated from Xinjiang Province, China [26]. The virus was propagated and titrated in Marc-145 cells grown in DMEM supplemented with 2% FBS. Poly (I:C)-LMW and poly (I:C)-HMW were purchased from InvivoGen (San Diego, CA, USA). 2′3′-cGAMP and polydA:dT were bought from InvivoGen (Hong Kong, China).

### 2.2. Subcloning of Porcine Innate Immune Signaling Adaptor Gene ORFs

The nine porcine adaptors [27,28] we previously constructed in the pEGFP-N1/C1 vectors were double-digested and ligated into the corresponding sites of pmCherry-N1/C1 vectors. The digested MyD88 (*Bgl*II/*EcoR*I), TRIF (*EcoR*I/*BamH*I), MAVS (*Bgl*II/*Kpn*I), RIPK2 (*Bgl*II/*EcoR*I), CARD9 (*Bgl*II/*Kpn*I), BCL10 (*Bgl*II/*EcoR*I), MALT1 (*Xho*I/*Kpn*I) and STING (*Bgl*II/*EcoR*I) DNA fragments were cloned into pmCherry-C1 and the expressed proteins carried an N-terminal red fluorescent protein (mCherry). The *Bgl*II/*Kpn*I digested ASC DNA fragment was cloned into pmCherry-N1, and the expressed ASC was fused with a C-terminal mCherry.

### 2.3. Preparation of Polyclonal Antibody against N Protein of PRRSV

The coding regions of the PRRSV N gene were amplified by PCR from PRRSV cDNA using the primers listed in Table 1. The resulting fragment was then inserted into the *Nco*I and *Xho*I restriction sites of the pET-28a expression vector, creating the construct pET-28a-ORF7. The protein was expressed in recombinant bacteria DE3/BL21 after 3 h induction with 1 mM isopropyl-b-D-thiogalactopyranoside (IPTG) at 30 °C. The bacterial pellet was then resuspended in PBS and subjected to sonication and centrifugation. The soluble protein in the supernatant were mixed with loading buffer and run on SDS-PAGE. The gel containing the target protein was excised and processed according to the instructions of the Micro Protein PAGE Recovery Kit (Sangon Biotech, Shanghai, China). The purified protein was obtained and evaluated for purity and amount by SDS-PAGE. Mice were immunized with 20 μg purified N protein plus immune adjuvant (1:1) (QuickAntibody-Mouse5W, Biodragon, Beijing, China) three times, and the polyclonal serum antibody was isolated from eyeball blood. The experiment was carried out in strict accordance with the institutional animal care and use protocol of Yangzhou University.

### 2.4. Construction of Full-Length cDNA Clones of HP-PRRSV XJ17-5 Carrying EGFP

To generate an infectious clone of PRRSV carrying the EGFP sequence, the full-genome cDNA clone of the HP-PRRSV XJ17-5 isolate was firstly constructed by the similar strategy as we previously described [29]. Briefly, three overlapping fragments (F1, F2, and F3) spanning the full-length genome of XJ17-5 were generated by PCR amplification using the high fidelity Primestar Max DNA polymerase (TAKARA) and a set of primers (Table 2). The full-length XJ17-5 cDNA clone pACYC177-CMV-rXJ17-5 (rXJ17-5) was obtained by sequential cloning of three fragments using 4 unique restriction enzyme sites (*Pac*I, *Afl*II, *Asc*I and *Not*I). The rXJ17-5-EGFP clone was constructed by inserting the EGFP coding sequence between the non-structural and structural genes as previously described [29]. The *Asc*I-*Kpn*I-EGFP-*Bcl*I-*Bgl*II fragment (1.1kb) synthesized was ligated into the *Asc*I and *Bgl*II sites of pACYC177-XJ17-5-F3. The F3-EGFP fragment was cut from the positive and sequence confirmed pACYC177-XJ17-5-F3-EGFP by *Asc*I and *Not*I, and ligated into the same sites of pACYC177-XJ17-5-F1-F2 to generate full-length pACYC177-XJ17-5-EGFP (rXJ17-5-EGFP). The obtained full-length rXJ17-5-EGFP clone was transfected into BHK-21 cells using Lipofectamine 3000 reagent (ThermoFisher Scientific, Waltham, MA, USA) and cell culture supernatants obtained at 48 h post transfection were serially passaged on Marc-145 cells to rescue PRRSV rXJ175-EGFP. The real-time infection of rXJ17-5-EGFP virus was visualized by fluorescence microscopy.

### 2.5. RNA Interference by siRNA

All siRNAs targeting monkey TRIF (GenBank accession: NM_001130428.1), MAVS (GenBank accession: KC415016.1) and STING (GenBank accession: MF622060.1) were designed and synthesized by Invitrogen (ThermoFisher Scientific, Waltham, MA, USA) and the siRNA sequences are listed in Table 3. Marc-145 cells were plated into 24-well plates and the cells with 70–80% confluency were transfected with 30–200 nM of siRNA using Lipofectamine 2000 (ThermoFisher Scientific) according to the manufacturer’s instructions. To determine the efficiency of the knockdown, total RNA was extracted from the cells at 24 h post transfection, and endogenous mRNAs of TRIF, MAVS and STING were quantified by RT-qPCR. In parallel, cells were lysed, and proteins were measured by Western blotting for STING expression with polyclonal anti-STING rabbit antibody (1:2000) (Proteintech, Rosemont, IL, USA).

### 2.6. Quantitative Reverse Transcription Polymerase Chain Reaction (RT-qPCR)

Infected Marc-145 cells were washed three times with PBS, and the total RNAs were extracted using TRIzol reagent (Invitrogen, ThermoFisher Scientific). The extracted RNA was reverse transcribed into cDNA with HiScript^®^ 1st Strand cDNA Synthesis Kit (Vazyme, Nanjing, China) according to the manufacturer’s instructions, quantitative PCR (qPCR) reaction (20 μL) was carried out on a StepOne Plus real-time PCR system (Applied Biosystems, Foster City, CA, USA) using ChamQ Universal SYBR qPCR Master Mix (Vazyme) and regular qPCR amplification program. All the gene primers used for qPCR amplification are listed in Table 4 and cellular β-actin mRNA was measured as an internal reference. Relative mRNA expression was calculated using the 2^−^^ΔΔCt^ method.

### 2.7. Western Blot Analysis

Cells were collected and lysed in radio-immunoprecipitation assay (RIPA) buffer (50 mM Tris pH 7.2, 150 mM NaCl, 1% sodium deoxycholate, 1% Triton X-100), and the cellular proteins were separated by 15% SDS-PAGE and transferred onto polyvinylidene difluoride (PVDF) membranes. After being blocked with 5% skim milk for 1 h at room temperature, the membranes were incubated with either mouse anti-PRRSV N protein (N) antiserum (1:1000) or other primary antibodies. Membranes were washed three times with TBST, followed by incubation with HRP-conjugated goat anti-mouse or rabbit IgG (1:10,000, Transgen Biotech, Beijing, China) as the secondary antibody. The protein signal was visualized by Western blot imaging system (Tanon, Shanghai, China) using an ECL chemiluminescent detection system (Tanon) according to the manufacturer’s instructions.

### 2.8. Flow Cytometry

Marc-145 cells were transfected using Lipofectamine 3000 with various adaptor plasmids or stimulated with agonists, and then infected with PRRSV XJ17-5-EGFP. At different time points post infection, cells were collected and analyzed by FACSVerse flow cytometer (BD Biosciences) for EGFP and mCherry expressions. All samples were gated based on forward scatter (FSC) and side scatter (SSC) to gate out cellular debris or dead cells. The levels of EGFP and mCherry were detected using the wavelength pairs 488/525 nm and 510/600 nm, respectively. The final analysis and graphical output were performed using FlowJo software (Tree Star, Inc., Ashland, OR, USA).

### 2.9. Virus TCID50 Titration

Marc-145 cells grown in 96-well plates were infected with ten-fold serial dilutions of PRRSV samples. After 1 h incubation at 37 °C, the supernatants were replaced with fresh DMEM containing 2% FBS. Five days post infection, the cytopathic effect (CPE) characterized by clumping and shrinkage of cells was obviously visible in Marc-145 cells and the viral titers, expressed as 50% tissue culture infective dose (TCID50), were calculated according to the method of Reed–Muench.

### 2.10. Statistical Analysis

The data are presented as the means + standard deviations (SD) (*n* = 3). Statistical significance between multiple groups was determined by performing *ANOVA* analysis with GraphPad Prism 6.0 software. The *p* value of <0.05 was considered significant.

## 3. Results

### 3.1. The Antiviral Activities of Ectopic Porcine Innate Signaling Adaptor Proteins against PRRSV Infection

**Figure 1:**
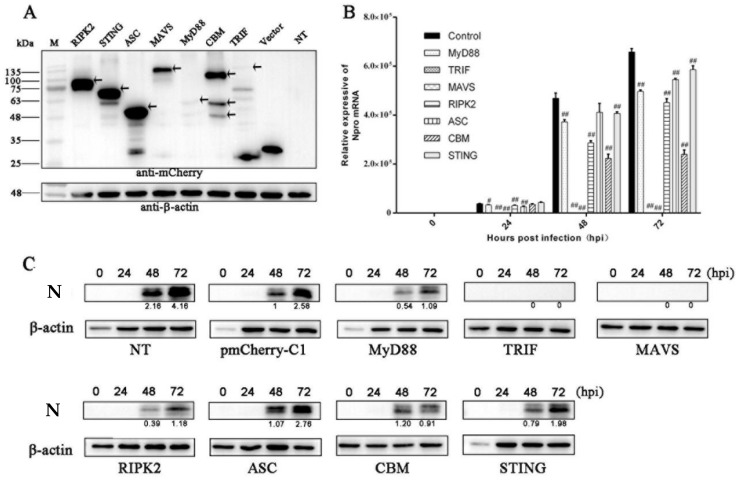
The expressions of the ectopic porcine innate signaling adaptor proteins and their effects on PRRSV infection. (**A**) Expressions of mCherry-tagged RIPK2, STING, ASC, MAVS, MyD88, CARD9-BCL10-MALT1, TRIF, together with vector (pmCherry-C1) in transfected 293T cells (0.5 μg each) were detected by Western blotting using anti-mCherry after transfection for 24 h. The β-actin was used as the internal control. The arrows mark the specific full length adaptor protein bands. NT denotes non-transfected. (B-C) Effects of ectopic porcine innate signaling adaptor proteins on PRRSV replication. Marc-145 cells seeded in 24-well plates were transfected with the ectopic porcine innate signaling adaptor-expressing plasmids or empty vector (0.25 μg each) using Lipofectamine 3000. At 24 h post transfection, the cells were infected with PRRSV at an MOI of 0.1, for 0, 24, 48, and 72 h respectively, and then assayed by RT-qPCR (**B**) and Western blotting (**C**). ^#^ *p* < 0.05, ^##^ *p* < 0.01 versus the vector control groups. The values of PRRSV N protein (N) quantification by gray scanning were shown below the bands following normalization by β-actin.

We previously cloned and characterized the nine porcine innate immune signaling adaptors which represent all currently known innate immune signaling pathways [27,28]. Here, we first examined the expression of the subcloned nine adaptor proteins (MyD88, TRIF, MAVS, RIPK2, ASC, CARD9, BCL10, MALT1 and STING) in transfected human embryonic kidney 293T cells. The Western blotting results show that all nine mCherry- tagged porcine adaptors are successfully expressed, with TRIF and MyD88 exhibiting low expression levels (Figure 1A). In Marc-145 cells, upon PRRSV infection, the nine adaptor gene transcriptions were all upregulated (Figure S1). To explore the impacts of nine adaptor proteins on PRRSV replication, the adaptor-transfected Marc-145 cells were infected with PRRSV-2 and the PRRSV replication was examined by measuring the viral N gene transcription using RT-qPCR and N protein expression using Western blotting. The RT-qPCR results show that the all the signaling adaptors decreased the expressions of PRRSV N mRNA in varying degrees at 24 h, 48 h and 72 h post infection, with the TRIF and MAVS as the most effective inhibitors of PRRSV replication (Figure 1B). The Western blotting results show that TRIF and MAVS are most effective in inhibition, MyD88, RIPK2, CBM and STING are intermediate in inhibition, and ASC is barely effective in inhibition of N protein expression at 48 h and 72 h post infection of PRRSV (Figure 1C).

### 3.2. The Antiviral Activities of Ectopic Porcine Innate Signaling Adaptor Proteins against PRRSV-EGFP Infection

**Figure 2:**
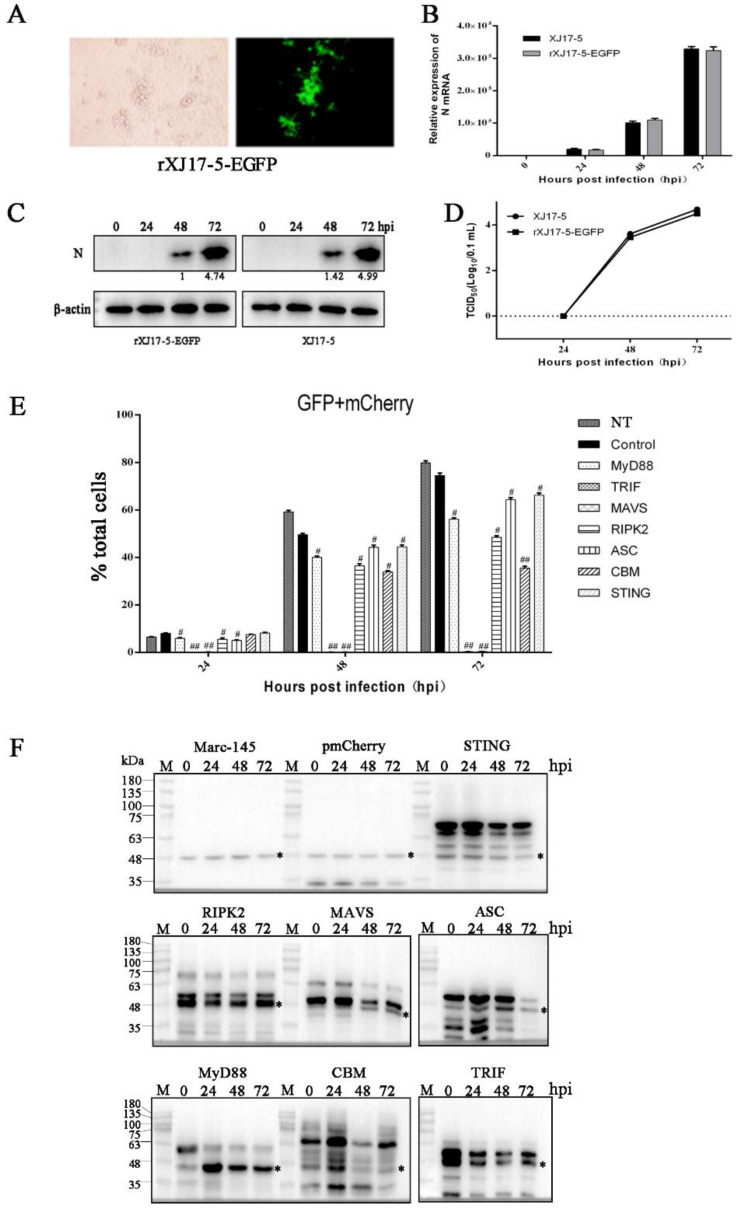
Identification of the rescued rXJ17-5-EGFP virus and the antiviral activities of ectopic porcine innate signaling adaptor proteins against PRRSV-EGFP. (**A**) Marc-145 cells were infected with PRRSV-EGFP at a MOI of 0.1. Cytopathic effects were observed and the GFP signal was visible under fluorescence microscope at 48 h post infection (hpi). (**B**–**D**) The in vitro growth kinetics of the cloned rXJ17-5-EGFP virus and the parental XJ17-5 virus. The growth curve of both PRRSVs in Marc-145 cells within 72 hpi were determined by RT-qPCR (**B**), Western blotting (**C**) and TCID50 assay (**D**). No significant difference was observed between the parental and cloned viruses. (**E**,**F**) Effects of ectopic porcine innate signaling adaptor proteins on PRRSV replication. Marc-145 cells were seeded into 24-well plates and transfected with the ectopic porcine innate signaling adaptor-expressing plasmid or empty vector control (0.25 μg each) using Lipofectamine 3000. At 24 h post transfection, the cells were infected with PRRSV-EGFP at an MOI of 0.1, for 0, 24, 48, and 72 hpi, and analyzed by flow cytometry (**E**) and by Western blotting (**F**). GFP signal represents the Marc-145 cells infected by PRRSV, whereas GFP/mCherry double signal represents the Marc-145 cells expressing adaptor proteins or vector mCherry and infected by virus. * represents the non-specific protein bands recognized by anti-mCherry antibody which can be used as internal controls. ^#^ *p* < 0.05, ^##^ *p* < 0.01 versus the vector control groups.

In order to better evaluate the antiviral effects of innate immune adaptor proteins, we obtained the GFP-labeled PRRSV virus rescued by reverse genetics and its replication can be measured by flow cytometry (Figure 2A). To ensure the effectiveness of the rescued PRRSV-EGFP, we compared the growth kinetics of the cloned PRRSV-EGFP with the parental PRRSV XJ17-5. As expected, PRRSV-EGFP and parental PRRSV showed comparable replication kinetics in Marc-145 cells as evidenced by N gene transcription (Figure 2B), N protein expression (Figure 2C) and TCID50 assay (Figure 2D). Next, the PRRSV replications in the presence of various adaptor proteins were examined by flow cytometry analysis of GFP signals in transfected and infected cells (raw data in Figure S2). The double positive signals of GFP and mCherry were collected to precisely reflect the PRRSV-EGFP replication in mCherry adaptor expressing cells (Figure 2E), and the results were identical to those of single GFP signals (not shown). At 48 h and 72 h post infection, substantial proportions of Marc-145 cells were infected by PRRSV-EGFP, accounting for 60–80% of total cells (Figure 2E and not shown). The seven adaptor proteins decreased the GFP signals in varying degrees relative to control cells, with TRIF and MAVS most effective (Figure 2E), similar to those results of N gene transcriptions. To ensure the adaptor protein expression in transfected Marc-145 cells, the mCherry-tagged adaptor expressions were detected by Western blotting, and the results show that all adaptors were expressed (Figure 2F). Among the 9 adaptors, the full length MASV, TRIF and RIPK2 did not appear likely due to the low-level expression or cleavage events during expression (Figure 2F). Marc-145 is a monkey cell line, whereas 293T is a human embryonic kidneys (HEK) cell line transformed with large T antigen. The results reflect the expression disparity in both primate cell types. Nevertheless, this did not impede the strong antiviral activities of MAVS, TRIF and RIPK2. Taken together, these results demonstrate that the seven innate adaptor signaling proteins are all likely involved in recognition, interaction and innate defense during PRRSV infection.

### 3.3. The Antiviral Activities of Endogenous TRIF, MAVS and STING Signaling against PRRSV Replication

**Figure 3:**
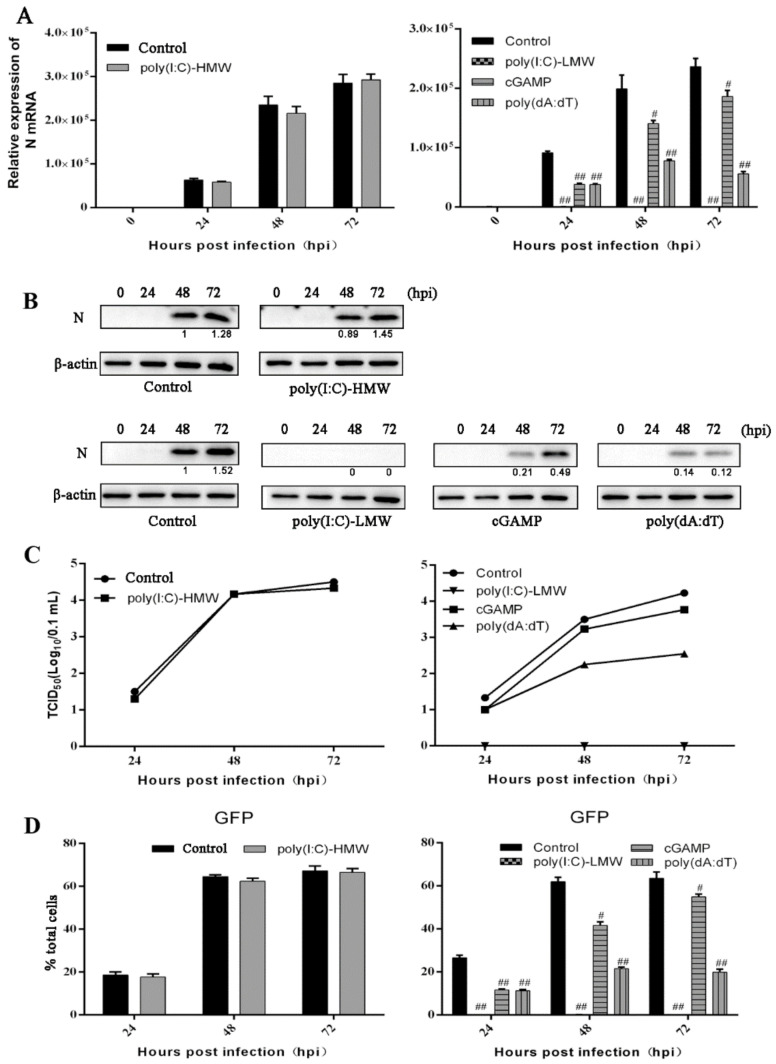
The effects of endogenous TRIF, MAVS and STING signaling on PRRSV replication. (**A**–**D**) Marc-145 cells grown in 24-well plates were mock stimulated (control) or stimulated with poly(I:C)-HMW addition (10 μg/mL) for TLR3-TRIF (left or top panels), transfection reagent transfection (control) or poly(I:C)-LMW transfection (1 μg/mL) for RIG-I/MDA5-MAVS, 2′3′-cGAMP transfection (1 μg/mL) for STING or poly(dA:dT) transfection (1 μg/mL) for cGAS-STING (right or bottom panels) for 24 h. Then the cells were infected with PRRSV at an MOI of 0.1, for 0, 24, 48, and 72 hpi, respectively, and assayed by RT-qPCR (**A**), Western blotting (**B**), TCID50 (**C**) and flow cytometry (**D**). ^#^ *p* < 0.05, ^##^ *p* < 0.01 versus the transfection control groups.

Based on the screening results and considering the importance of the nucleic acid-sensing signal in the antiviral activity, The nucleic acid signaling related adaptors TRIF, MAVS and STING were chosen and their endogenous signaling were investigated for antiviral roles in PRRSV infection. Several agonists were used to trigger the endogenous innate signaling, including the poly(I:C)-HMW for TLR3-TRIF, poly(I:C)-LMW for RIG-I/MDA5-MAVS, 2′3′-cGAMP for STING, and poly(dA:dT) for cGAS-STING. The poly(I:C)-HMW as the agonist for TLR3 when added to cells is able to specifically activate the downstream adaptor TRIF signaling, however, compared with non-stimulated control cells, there was no significant changes of PRRSV infection in poly(I:C)-HMW treated Marc-145 cells as shown by N gene transcription (left, Figure 3A) and N protein expression (top, Figure 3B), TCID50 assay (left, Figure 3C) and GFP flow cytometry (left, Figure 3D and Figure S3). On the other hand, compared with transfection control cells, transfection of poly(I:C)-LMW, 2′3′-cGAMP, and poly(dA:dT) obviously decreased the N gene transcription (right, Figure 3A) and N protein expression (bottom, Figure 3B), virus titers in TCID50 assay (right, Figure 3C) and GFP signals in flow cytometry (right, Figure 3D and Figure S3). These results indicate that endogenous MAVS and STING and not TRIF in Marc-145 cells can inhibit PRRSV replication.

### 3.4. Knockdown of Endogenous TRIF, MAVS and STING Enhances PRRSV Replication

**Figure 4:**
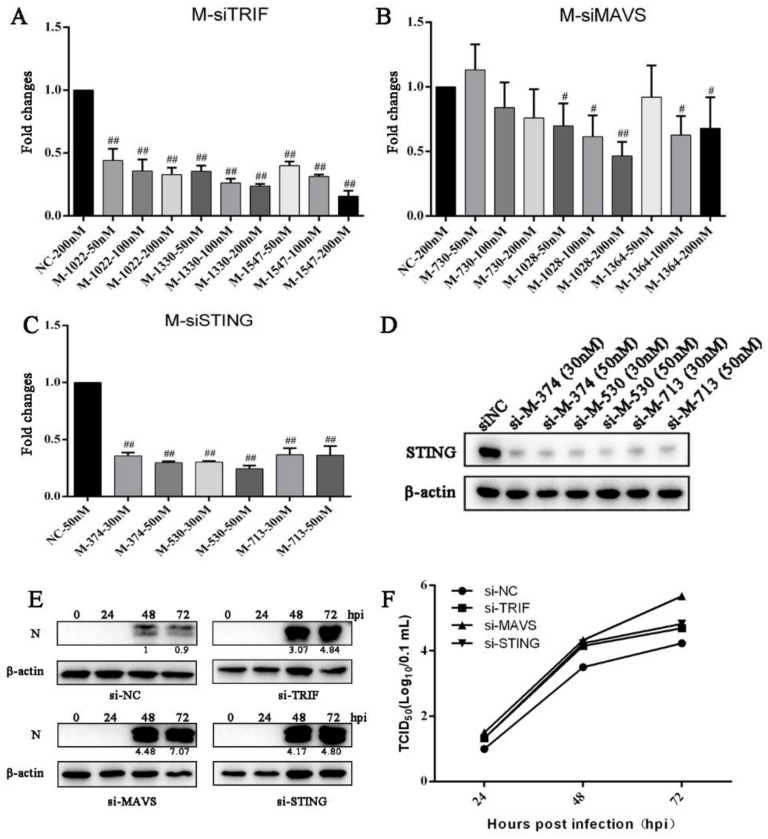
Knockdown of endogenous TRIF, MAVS and STING and their effects on PRRSV replication. (**A**–**D**) After transfection of Marc-145 cells with siTRIF, siMAVS, siSTING or siNC (negative control siRNA) at the indicated concentrations for 48 h, TRIF, MAVS and STING gene expressions were quantified by RT-qPCR and Western blotting. (**E**) Marc-145 cells were treated by transfection with either siNC, siTRIF, siMAVS or siSTING for 48 h, followed by infection with PRRSV at an MOI of 0.1. Cells were harvested at 0, 24, 48, and 72 hpi, and PRRSV N proteins (N) were detected by Western blotting. (**F**) Viral titers in the supernatants were assayed by TCID50. ^#^ *p* < 0.05, ^##^ *p* < 0.01 versus the siNC groups.

To further explore the roles of endogenous TRIF, MAVS and STING in the PRRSV replication, the siRNAs were used to knockdown the TRIF, MAVS and STING in Marc-145 cells before PRRSV infection. Three siRNA duplexes were designed for TRIF, MAVS and STING, and knockdown efficiency was examined by RT-qPCR (Figure 4A–D). The siRNAs with high knockdown efficiency were selected for TRIF (siRNA 1330) (Figure 4A), MAVS (siRNA 1028) (Figure 4B) and STING (siRNA 530) (Figure 4C,D). In the TRIF, MAVS and STING siRNA-treated Marc-145 cells, the PRRSV N protein expressions were substantially increased at 48 h and 72 h post infection relative to control siRNA-treated cells (Figure 4E). Similarly, compared with control siRNA-treated cells, the PRRSV titers were obviously heightened in TRIF, MAVS and STING siRNA-treated cells at 24 h, 48 h and 72 h post infection (Figure 4F). These results suggest that endogenous TRIF, MAVS and STING are all part of the host defense mechanism, necessary for the inhibition of PRRSV replication.

### 3.5. Innate TLR3-TRIF Signaling Plays a Differential Role in Marc-145 Cells and Porcine Macrophages

**Figure 5:**
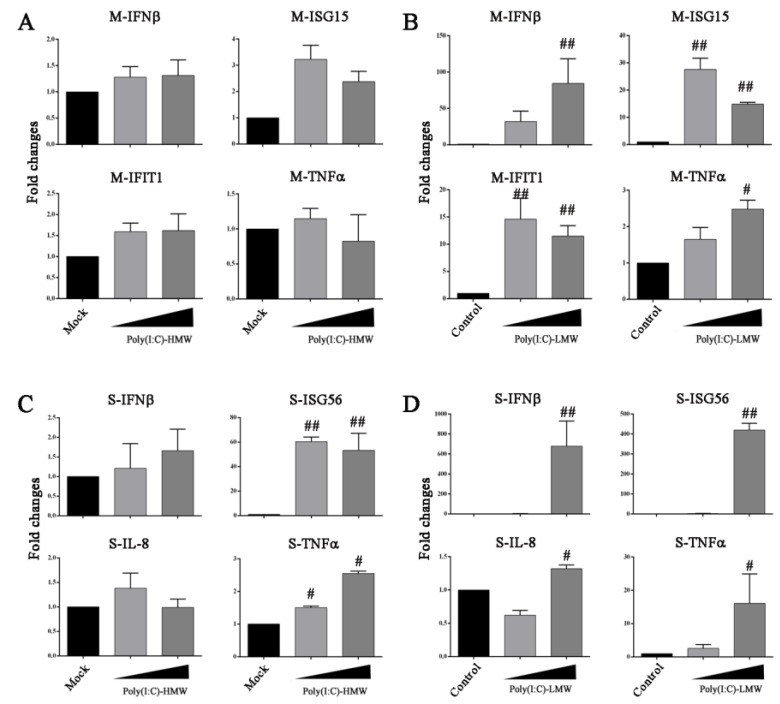
Detection of the differential responses of Marc-145 cells and porcine macrophages to poly(I:C). (**A**,**B**) Marc-145 grown in 24-well plates were stimulated with poly(I:C)-HMW addition (10 and 20 μg/mL) or poly(I:C)-LMW transfection (0.5 and 1 μg/mL) for 24 h. (**C**,**D**) PAMs (3D4/21) grown in 24-well plates were stimulated with poly(I:C)-HMW addition (10 and 20 μg/mL) or poly(I:C)-LMW transfection (0.5 and 1 μg/mL) for 24 h. The harvested cells were analyzed by RT-qPCR for downstream gene expressions as indicated. ^#^ *p* < 0.05, ^##^ *p* < 0.01 versus the mock or transfection control groups.

Due to the discrepancy of endogenous TRIF in the inhibition of PRRSV replication, we wondered whether there is a defect of poly(I:C)-HMW-TLR3 signaling in Marc-145 cells. The poly(I:C)-HMW was added to the Marc-145 cells to trigger TLR3-TRIF signaling and the downstream gene transcriptions were examined by RT-qPCR. In parallel, the poly(I:C)-LMW was transfected into Marc-145 cells to trigger RLR (RIG-I/MDA5)-MAVS signaling and the downstream gene transcriptions were examined. Indeed, no obvious downstream gene transcriptions of IFNα, IFNβ, ISG15, IFIT1, IL-8 and TNFα were observed in poly(I:C)-HMW-treated Marc-145 cells (Figure 5A and data not shown), whereas the gene transcriptions of IFNα, IFNβ, ISG15 and IFIT1 were clearly induced in poly(I:C)-LMW-treated Marc-145 cells (Figure 5B and data not shown). On the other hand, porcine macrophages (3D4/21 cells) were also stimulated with poly(I:C)-HMW and poly(I:C)-LMW, and in both cases, the downstream ISG56 gene transcriptions were clearly activated (Figure 5C,D). The results suggest that the inability of poly(I:C)-HMW to trigger the anti-PRRSV activity is due to the defect of TLR3 signaling in Marc-145 cells.

## 4. Discussion

PRRS is an economically important viral disease of swine all over the world; at present, because of the high genomic diversity, antigenic heterogeneity and antibody-dependent enhancement of PRRSV infection, vaccination strategies are obviously ineffective for controlling PRRSV infection [30]. The complex interaction between PRRSV and host immune response has not been fully understood [31]. PSSRV exhibited extensive and intensive suppression of innate immunity, meanwhile this innate immunity exerts an important role in defense against PRRSV [14,16]. However, the innate immunity comprises dozens of signaling pathways and it is difficult to systematically dissect all innate signaling pathways for antiviral activity against PRRSV. We previously characterized nine porcine innate immune signaling adaptors which represent all currently known TLR, RLR, NLR, CLR and CDR innate signaling pathways [27,28]. In this study, we expressed the nine porcine signaling adaptors to mimic five families of innate immune signaling pathways and found that the seven signal adaptors can inhibit PRRSV infection of Marc-145 cells to varying degrees. Among the seven adaptors, most effective TRIF, MAVS and STING were selected and the three endogenous adaptors were further confirmed to be necessary for anti-PRRSV activity. This suggests that multiple porcine PRR signaling pathways might be involved in the recognition of and defense against PRRSV, which are more than previously appreciated TLR3/7/8/9 and RLRs [25].

TLR signaling is broadly divided into MyD88 and TRIF dependent signaling pathways. All TLRs except TLR3 utilize MyD88 as the signaling adaptor to relay NF-κB signaling, whereas TLR3 and TLR4 utilize TRIF as signaling adaptors and TRIF is the only signaling adaptor for TLR3 [32,33]. In this study, ectopic MyD88 exhibited intermediate anti-PRRSV activity, reflecting previously reported roles of several TLRs in the defense against PRRSV [34]. Ectopic TRIF showed most effective anti-PRRSV activity and knockdown TRIF in Marc-145 enhanced the PRRSV replication, suggesting the critical role of TRIF in anti-PRRSV infection. However, the TLR3 agonist poly(I:C)-HMW did not induce appreciable anti-PRRSV activity in Marc-145 cells, indicating the existence of a defect in TLR3 signaling in Marc-145 cells which was confirmed by the failure of downstream gene induction. The defect of TLR3 signaling in Marc-145 cells may be one reason that this cell type is permissive for PRRSV replication, which is reminiscent of another cell type, human hepatoma Huh7.5, with defects in RIG-I and other receptor signaling proteins that make it highly permissive to HCV infection [35]. In contrast, in porcine macrophages 3D4/21 cells, poly(I:C)-HMW is able to induce downstream gene transcriptions. Marc-145 is a non-human primate cell line, which is from African green monkey cell line MA-104. The disparity of Marc-145 cells and porcine macrophages in the sensitivity to swine IFNβ and PKR inhibitor was previously reported [36], and more disparities can be further discovered in the future.

RLRs play a critical role in sensing RNA infections by recognition of the cytosolic double stranded RNA from virus infections. Similarly, our results demonstrate that MAVS, which mediates RLR signaling, plays a critical role against PRRSV infection: Both expression of MAVS and poly(I:C)-LMW stimulation exhibited effective anti-PRRSV activity, whereas knockdown of MAVS enhanced PRRSV replication. In fact, the important role of MAVS in PRRSV infection has been previously shown by several studies: The production of zinc finger anti-PRRSV protein, ZAP, was dependent on MAVS [20]; IFI16 exerted anti-PRRSV effects in a MAVS-dependent manner [23]. On the other hand, MAVS was targeted for cleavage and degradation by PRRSV nsp2 and nsp11, respectively, for immune evasion [37,38]. Therefore, RLR-MAVS seems have a central role in the sensing of, and defense against, PRRSV infection. The CDR cGAS-STING pathway is mainly involved in sensing DNA viruses, but it is also shown to sense RNA infection to supplement RNA sensors during RNA infections [39,40]. Here our results demonstrate that expression of STING and agonist stimulations induced intermediate anti-PRRSV activity, whereas knockdown of STING enhanced PRRSV replication. Therefore, the role of the cGAS-STING signaling pathway in PRRSV infection warrants further investigation in particular in porcine macrophages in the future.

NLR signaling adaptor RIPK2 is involved in the activation of NF-κB and MAPK [41], and another NLR adaptor ASC mediates inflammasome formation, even though ectopic ASC is a weak NF-κB activator [42]. The CLR pathway signal is transmitted by the adaptor CBM complex composed of CARD9, BCL10 and MALT1, which activates downstream NF-κB [43]. Here, we found that ectopic RIPK2 and CBM complex both conferred intermediate anti-PRRSV activities. Although we did not explore the roles of endogenous RIPK2 and CBM against PRRSV infection, the anti-PRRSV activities by these two types of adaptors in vivo cannot be neglected based on our results. Given that RIPK2, ASC and CMB mainly play roles in NF-κB and inflammasome mediated inflammation, it is reasonable that these adaptors have more important roles in PRRS pathogenesis than in anti-PRRSV infection.

In conclusion, our data show for the first time that the nine innate signaling adaptor proteins could inhibit PRRSV infection in varying degrees, which is helpful to understand the host antiviral response against PRRSV infection and design anti-PRRSV therapeutics.

## Figures and Tables

**Table 1:** PCR primers used for the construction of recombinant pET-28a-N.

Primer Names	Primer Sequences (5′-3′)
PRRSV-N-F	CATGCCATGGATGCCAAATAACAACGGCAAGC
PRRSV-N-R	CCGCTCGAGTACTGAGGGTGATGCTGTGGC

Note: the restriction enzyme sites are underlined.

**Table 2:** PCR primers used for the construction of XJ17-5 infectious clones.

Primer Names	Primer Sequences (5′-3′) *
XJ17-5 -*Pac*I-F1	AGCTCGTTAATTAATACATGACGTATAGGTGTTGGCT
XJ17-5 -*Afl*II-R1	CATAGGTGCTTAAGTTCATTACCACCTGTAACGGAT
XJ17-5 -*Afl*II-F2	ATCCGTTACAGGTGGTAATGAACTTAAGCACCTATG
XJ17-5 -*Asc*I-R2	CCTTTCTGGCGCGCCCGAAAC
XJ17-5 -*Asc*I-F3	GTTTCGGGCGCGCCAGAAAGG
XJ17-5 -1R3	***AGCGAGGAGGCTGGGACCAT****GCCGGCC*TTTTTTTTT TTTTTTTTTTTTAATTACGGCCGCATGGTTCT
XJ17-5 -*Not*I-2R3	ACAGGCGGCCGC*GTCCCATTCGCCATTACCGAGGGG* *ACGGTCCCCTCGGAATGTTGCCCAGCCGGCGCC**AGC*** ***GAGGAGGCTGGGACCAT*** ^#^

* The unique restriction enzyme sites used for cloning purposes were shown as underlined. ^#^ The hepatitis D virus ribozyme sequence was shown in italic and the overlapped region was highlighted in bold.

**Table 3:** The siRNA sequences for monkey TRIF, MAVS, STING genes.

siRNA Name	Primer Sequences (5′-3′)
TRIF-1022-F	CCACCUCUCCAAAUACCAATT
TRIF-1022-R	UUGGUAUUUGGAGAGGUGGTT
TRIF-1330-F	GCGAUAGACCACUCAGCUUTT
TRIF-1330-R	AAGCUGAGUGGUCUAUCGCTT
TRIF-1547-F	CCCAGAUCUUCGCUAGGAATT
TRIF-1547-R	UUCCUAGCGAAGAUCUGGGTT
MAVS-730-F	GCAGGGUCAGUUGUAUCUATT
MAVS-730-R	UAGAUACAACUGACCCUGCTT
MAVS-1028-F	CCAAUCCAGCACCAUCCAATT
MAVS-1028-R	UUGGAUGGUGCUGGAUUGGTT
MAVS-1364-F	GCCCAGAGGAGAAUGAGUATT
MAVS-1364-R	UACUCAUUCUCCUCUGGGCTT
STING-374-F	GCCUUUCGCAGGCACUGAATT
STING-374-R	UUCAGUGCCUGCGAAAGGCTT
STING-530-F	CCCGGAUUCAAACUUACAATT
STING-530-R	UUGUAAGUUUGAAUCCGGGTT
STING-713-F	GGGUUUACAGCAACAGCAUTT
STING-713-R	AUGCUGUUGCUGUAAACCCTT
NC-F	UUCUCCGAACGUGUCACG UTT
NC-R	ACGUGACACGUUCGGAGAATT

Note: NC denotes negative control siRNA sequences.

**Table 4:** Primers for RT-qPCR in this study.

Primer Names	Primer Sequences (5′-3′)
PRRSV N-F	ATAACAACGGCAAGCAGCAG
PRRSV N-R	CTCTGGACTGGTTTTGTTG
M-MyD88-F	TCTCTCCAGGTGCCCATCAGAAG
M-MyD88-R	GCAAGGCGAGTCCAGAACCAAG
M-TRIF-F	AGTCGCCACTGCAACTTTCTGTAG
M-TRIF-R	GGCTGATGAAGGAGGAGGAGGAG
M-MAVS-F	CCCGAAAGTGCCTGCCAACC
M-MAVS-R	TTGGATGGTGCTGGATTGGTGAAC
M-RIPK2-F	CCCTGCCAGTTCCTCAAGACAATG
M-RIPK2-R	AGCCCTTTGAGACGCAGAAATGG
M-ASC-F	GATGCCATCGACCTCACTGACAAG
M-ASC-R	TCCGCCACCTCCTTCATGCC
M-CARD9-F	ACTTCCACTTCCGTCTGCCTCTC
M-CARD9-R	CCACAGTCCCGCCAGTCCTC
M-BCL10-F	CCAGATGGAGCCACGAACAACC
M-BCL10-R	GGATGGTACATGACAGTGGATGCC
M-MALT1-F	CGAGGTTTGGGAAGGAAGACTTGC
M-MALT1-R	GTGATAATGCCCTGCTGCTCCTG
M-STING-F	TCGCTGTCCCAAGAGGTTCTCC
M-STING-R	GGGCACCGCTGAGTTCTTCAAG
M-β-actin-F	AGAAGATGACCCAGATCATGTTTGA
M-β-actin-R	TCCATCACGATGCCAGTGGTA
M-IFN-β-F	ATGGAGAAAGGACATTCTAACTGCAA
M-IFN-β-R	AGAAGCACAACAGGAGAGCAATTT
M-ISG15-F	CTCTGAGCATCCTGGTGAGGAA
M-ISG15-R	CGAAGGTCAGCCAGAACAGGT
M-IFIT1-F	TCAGGTCAAGGATAGTCTGGAGCA
M-IFIT1-R	TGTCTAGGAATTCAATCTGGTCCAA
M-TNF-α-F	CATGTTGTAGCAAACCCTCAAGC
M-TNF-α-R	ATGGCACCACCAGCTGGTTAT
S-IFN-β-F	TGAGCATTCTGCAGTACCTGA
S-IFN-β-R	CCGGAGGTAATCTGTAAGTCTGT
S-ISG56-F	ATGGGAGTTGGTCATTCAAGA
S-ISG56-R	CAGGTGTTTCACATAGGCCA
S-IL-8-F	CTGCAGTTCTGGCAAGAGTAAGT
S-IL-8-R	CACTCTCAATCACTCTCAGTTCCT
S-TNF-α-F	ATCGCCGTCTCCTACCAGA
S-TNF-α-R	TCGATCATCCTTCTCCAGCT
S-β-actin-F	ATGAAGATCAAGATCATCGCG
S-β-actin-R	TCGTACTCCTGCTTGCTGATC

Note: M denotes monkey, and S denotes swine.

## Data Availability

The data presented in this study are available in insert article.

## Supplementary Materials

The following are available online at https://www.mdpi.com/article/10.3390/vaccines9101176/s1, Figure S1 Detecting the expressions of the nine adaptor gene transcriptions in Marc-145 cell infected with PRRSV; Figure S2 Effects of ectopic porcine innate signaling adaptor proteins on PRRSV replication; Figure S3 Effects of endogenous TRIF, MAVS and STING signaling against PRRSV replication; and the raw data of Western blotting.
