# Supplementary material for: Screening of Porcine Innate Immune Adaptor Signaling Revealed Several Anti-PRRSV Signaling Pathways
Source: Vaccines (Basel). 2021 Oct 14;9(10):1176. doi: 10.3390/vaccines9101176 (PMC8538207; doi:10.3390/vaccines9101176)
Supplement: Supplementary file 1 [file vaccines-09-01176-s001.zip › vaccines-1343713-supplementary.pdf]

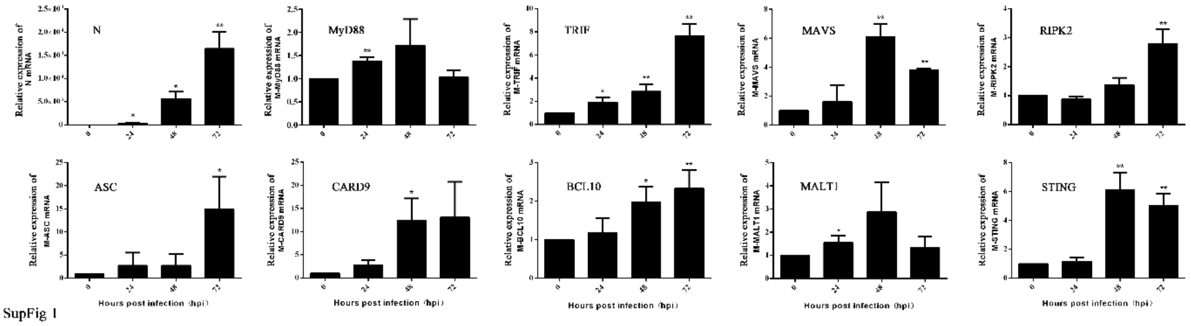

SupFig 1

Figure S1. Detecting the expressions of the nine adaptor gene transcriptions in Marc-145 cell infected with PRRSV. Marc-145 cells seeded in 24-well plates were infected with PRRSV at a MOI of 0.1, incubated for 0, 24, 48, and 72 h. The harvested cells were analyzed by RT-qPCR for the transcription of PRRSV N protein (N), MyD88, TRIF, MAVS, RIPK2, ASC, CARD9, BCL10, MALT1 and STING genes. * $p < 0.05$; ** $p < 0.01$.

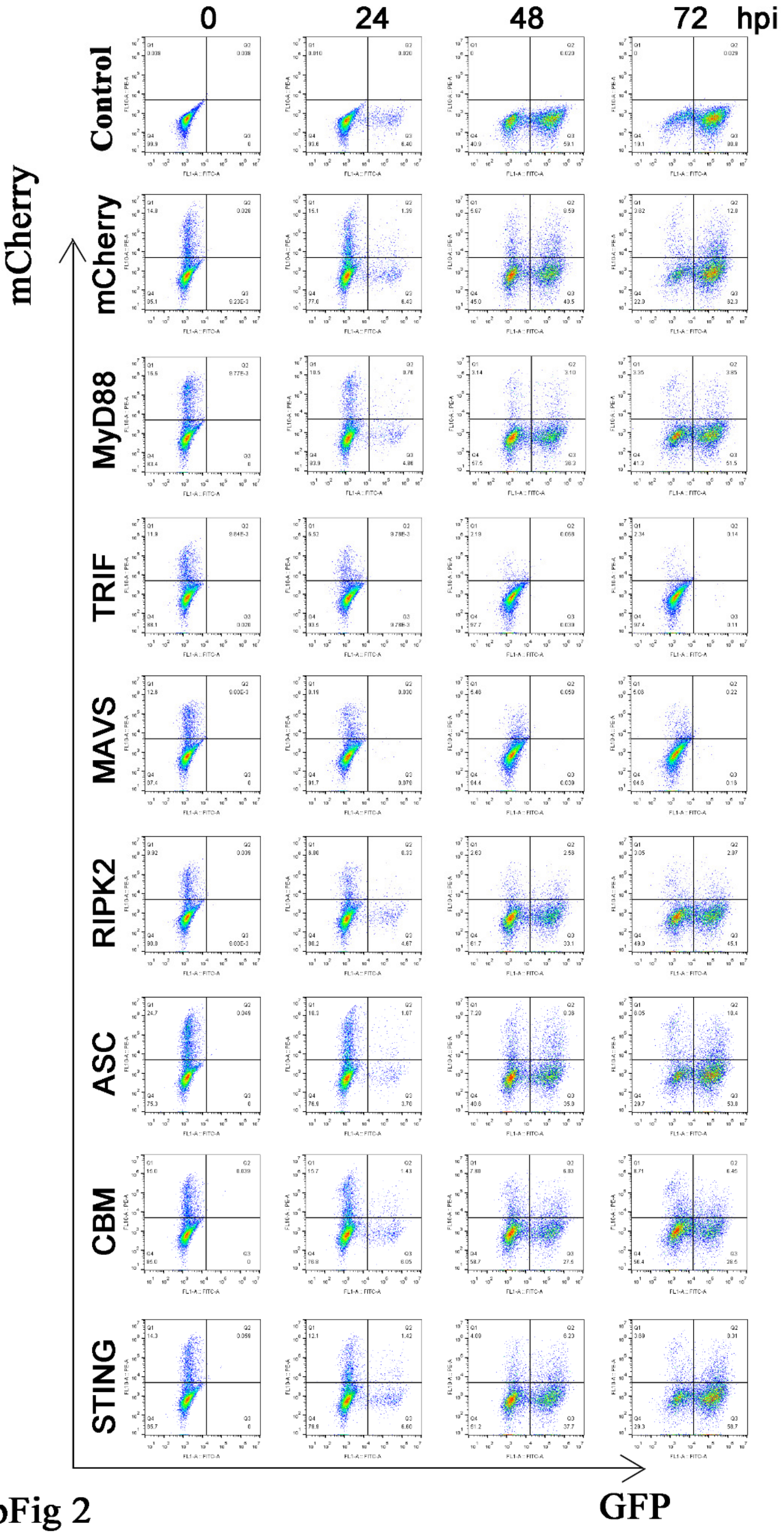
0
24
48
72
hpi
Control
mCherry
MyD88
TRIF
MAVS
RIPK2
ASC
CBM
STING
mCherry
GFP

SupFig 2

Figure S2. Effects of ectopic porcine innate signaling adaptor proteins on PRRSV replication. Marc-145 cells seeded in 24-well plates were transfected with the ectopic porcine innate signaling adaptor-expressing plasmids or empty vector (0.25 μg each) by Lipofectamine 3000. At 24 h post transfection, the cells were infected with PRRSV at an MOI of 0.1, for 0, 24, 48, and 72 h, and analyzed by flow cytometry. GFP signal represents the Marc-145 cells infected by PRRSV, whereas GFP/mCherry double signal represents the Marc-145 cells expressing adaptor proteins and infected by PRRSV.

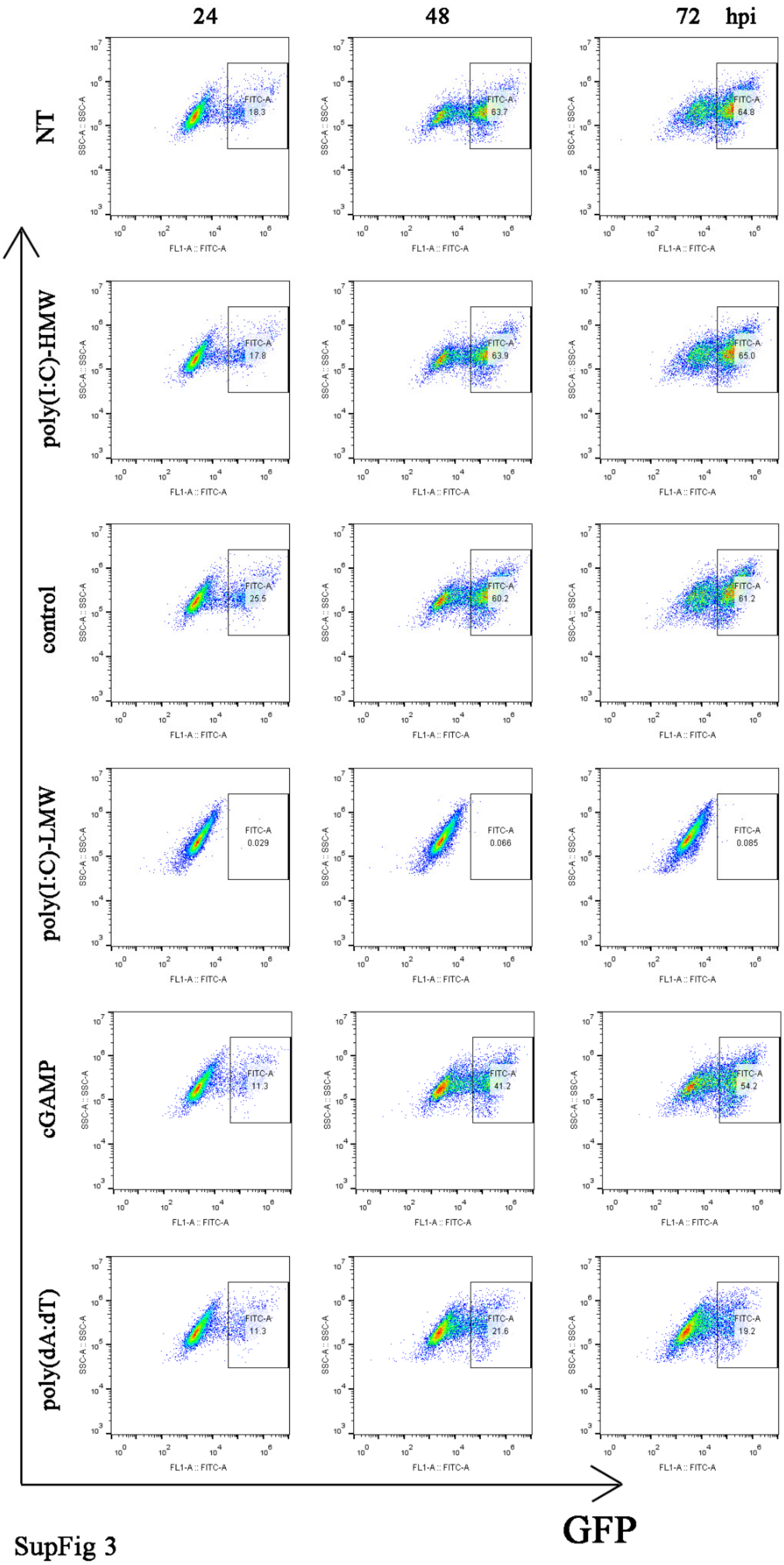
24
48
72 hpi
NT
poly(I:C)-HMW
control
poly(I:C)-LMW
cGAMP
poly(dA:dT)
FITC-A 18.3
FITC-A 63.7
FITC-A 64.8
FITC-A 17.8
FITC-A 63.9
FITC-A 65.0
FITC-A 25.5
FITC-A 60.2
FITC-A 61.2
FITC-A 0.029
FITC-A 0.066
FITC-A 0.085
FITC-A 11.3
FITC-A 41.2
FITC-A 54.2
FITC-A 11.3
FITC-A 21.6
FITC-A 19.2
SSC-A :: SSC-A
FL1-A :: FITC-A
GFP

SupFig 3

Figure S3. Effects of endogenous TRIF, MAVS and STING signaling against PRRSV replication. Marc-145 cells grown in 24-well plates were stimulated with poly(I:C)-HMW (10μg/mL) addition for TRIF, poly(I:C)-LMW (1μg/mL) transfection for MAVS, 2′3′-cGAMP (1μg/mL) transfection for STING or poly(dA:dT) (1μg/mL) transfection for cGAS-STING for 24 h. Next, the cells were infected with PRRSV at an MOI of 0.1, for 0, 24, 48, and 72 h, and then assayed by flow cytometry. GFP signal represents the Marc-145 cells infected by PRRSV.
